# Emergency Room Burden of Cardiac Complications in California

**DOI:** 10.7759/cureus.70834

**Published:** 2024-10-04

**Authors:** Lydia Wilson, Sasha Singh, Daryoush Javidi

**Affiliations:** 1 Medicine, California University of Science and Medicine, Colton, USA; 2 Medical Education, California University of Science and Medicine, Colton, USA

**Keywords:** burden of cardiovascular diseases, cardiovascular disease, emergency service, lack of access to primary care, southern california

## Abstract

Introduction

San Bernardino County has higher age-adjusted death rates due to cardiovascular disease (CVD) and a higher ratio of patients to primary care physicians when compared to the rest of California. With these trends, it is important to consider the impact on emergency departments (EDs). This study examines the burden of cardiac complications on EDs in San Bernardino County and across California. Additionally, it examines the difference in cardiac-related ED burden between hospitals inside and outside of primary care shortage areas (PCSAs).

Methods

Data were obtained from the California Health and Human Services Open Data Portal. Baseline comparisons used the calculated averages and percentage of ED visits that were cardiac-related. Further analysis was done using independent samples t-tests on IBM SPSS Statistics 28.0.1.0 (IBM Corp., Armonk, NY), checking for statistically significant differences in ED burden between PCSA and non-PCSA hospitals and between different geographical regions of California.

Results

Independent samples t-tests showed that rates of cardiac-related ED visits do not differ by PCSA designation in San Bernardino (p > 0.05), but they are significantly higher in PCSA hospitals when looking at Southern California and California (p < 0.05). Additionally, rates of cardiac-related ED visits seen in San Bernardino were significantly higher than in the rest of Southern California and California (p < 0.001).

Conclusion

Higher rates of cardiac-related ED visits in PCSAs of Southern California and California indicate a need to address issues of primary care access and enhance preventive measures for CVD. Furthermore, the increased ED burden of cardiac cases in San Bernardino County highlights the requirement for localized intervention.

## Introduction

By 2035, it is projected that 45.1% of the United States population will have at least one diagnosis of hypertension, coronary artery disease, heart failure, stroke, or atrial fibrillation, which would be a 30% increase in the total population with some form of cardiovascular disease (CVD) compared to 2015 [1]. In California, 5.8% of adults reported having some form of heart disease in 2016 [2]. With this already high and continuously increasing prevalence of CVD, it is important to consider the impact on the healthcare system.

From 2006-2014, there were 2.9 million patients admitted to hospitals across the United States for cardiac arrest, and 50% of these were cases of primary cardiac arrest not due to a known underlying cause [3]. However, more than 40% of cardiac arrest patients die in the emergency department (ED) and are, therefore, not included in hospital admission data [3]. This indicates that the burden of cardiac conditions on ED case load is significantly higher than can be inferred from admission data and is an area that needs to be studied further.

San Bernardino County is the largest county in the continental United States by land area and has a population of just over two million people [4]. In 2020, 7% of the population in San Bernardino County was diagnosed with heart failure, which is lower than that of neighboring counties [5]. However, the risk-adjusted prevalence of hospitalizations in San Bernardino County for heart failure was 341.2 per 100,000 residents compared to the statewide prevalence in California of 297.2 per 100,000 residents [5]. Additionally, in 2017, the age-adjusted death rate due to CVD in San Bernardino County was 184.7 per 100,000 residents compared to California’s state average of 142.9 [6].

One component that is likely contributing to these high rates of CVD in San Bernardino County is a lack of access to primary care physicians. In the county, there are 1,747 people for every primary care physician, which is higher than the national target ratio of 1,050 patients per primary care physician [6]. While there may be areas within the county that have adequate physician-to-patient ratios, there are likely many areas with a severe shortage of access to preventive care.

A lack of access to primary care physicians is associated with higher mortality, and patients without established providers have been shown to have worse health outcomes, including more comorbidities and advanced disease [7]. Also, approximately 60% of excess coronary heart disease events in populations with low socioeconomic status, such as San Bernardino County, were attributable to factors beyond traditional risk factors like smoking and high blood pressure [8]. Some of this may be attributed to the lack of access to primary care providers, but it is important to note that nearly 90% of US county populations in primary care shortage areas (PCSAs) had additional social determinants of health markers [9].

With the increasing prevalence of cardiac conditions, it is important to consider the impact that this has on the ED burden. Of note, over the past decade, the number of EDs in California has decreased by 4% while the number of ED visits has risen by 7.4%, leading to significantly longer wait times [10]. Higher volume in the ED can harm both the patients and physicians. For example, there may be more risks involved in discharge decisions when beds are full in the ED [11]. Additionally, ED physicians often experience a high cognitive load due to the inherent nature of working in acute care settings, and cognitive overload has been associated with unlearning, mental burnout, and patient errors [12]. Because of this, it is imperative to consider what conditions are contributing most to the ED caseload and find ways to address and improve this.

In this study, we considered the cardiac burden in the EDs of San Bernardino County compared to the rest of Southern California, as well as to the whole state of California. We also evaluated the difference in this burden between PCSA and non-PCSA hospitals to depict the impact that access to primary care may have on managing cardiac conditions and preventing exacerbations.

## Materials and methods

We obtained our data from the California Health and Human Services Agency (CalHHS) Open Data Portal, using the public and de-identified dataset, “Emergency Department Volume and Capacity” [13]. This dataset provides the number of all ED visits in 2021 for 251 hospitals in California. It also categorizes the visits as active COVID-19 infections, mental health-related, stroke, chronic obstructive pulmonary disease (COPD), history of COVID-19, hypertension, obesity, diabetes, cardiac-related, sepsis, cancer, substance abuse, homelessness, pneumonia, respiratory, or asthma. Any patients that did not fall into one of these categories were accounted for in the number of total ED visits.

We narrowed the scope of the data to focus only on ED visits classified as primarily cardiac-related. We then designated hospitals as being in Southern California if they were in the Los Angeles, Imperial, Riverside, Orange, San Bernardino, Santa Barbara, San Diego, or Ventura counties [14].

Using the numbers of cardiac-related ED visits and total ED visits provided in the dataset, we calculated the frequency of cardiac-related ED visits for each hospital. For counties with data from multiple hospitals, we averaged the frequencies of cardiac-related ED visits. This allowed us to obtain an initial characterization of the geographic distribution of the burden of cardiac-related cases in EDs across California. With the use of the website Datawrapper (Datawrapper GmbH, Berlin, Germany), we created a heatmap of the state of California that depicts this distribution. Given our focus on San Bernardino County, as well as the vast disparities in access to healthcare and determinants of health across the county, we chose to also compare frequencies for all hospitals in San Bernardino County that reported data.

In the dataset utilized, hospitals were designated as being in a PCSA based on the California Healthcare Workforce Policy Commission, which uses population and poverty data from the United States Census Bureau [15]. To compare the effects of primary care shortages on cardiac-related ED visits, we performed three separate independent sample t-tests that evaluated the difference in rates based on PCSA status between hospitals within San Bernardino County, within Southern California, and within California.

Finally, in comparing the overall ED burden of cardiac cases by geographic region, we performed two independent sample t-tests. The first compared the frequencies of cardiac-related ED visits in San Bernardino County with the rest of Southern California, while the second compared San Bernardino County with the rest of California.

For all statistical analyses, we utilized IBM SPSS Statistics 28.0.1.0 (IBM Corp., Armonk, NY) and considered a p-value of <0.05 to be significant.

## Results

**Figure 1:**
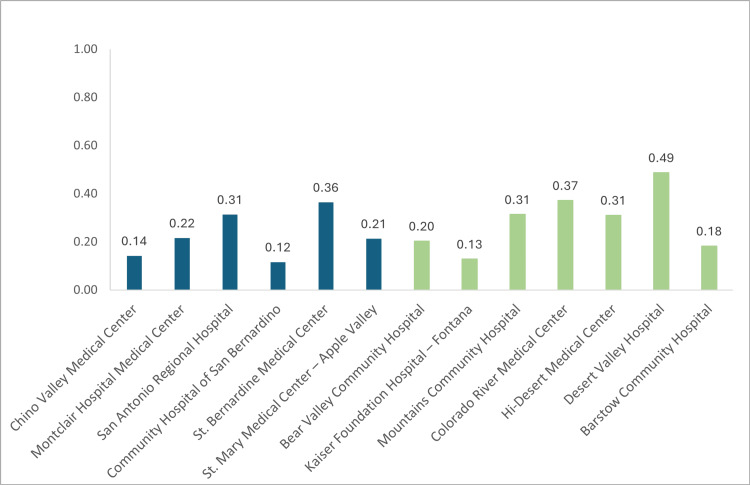
Percentage of ED visits that are cardiac-related in San Bernardino County Average percentages of ED visits that were related to cardiac complications in San Bernardino, 2021, separated by hospital and indication of primary care shortage area. Green corresponds to hospitals in primary care shortage areas. Blue corresponds to hospitals in non-primary care shortage areas.

From this dataset, hospitals in San Bernardino County that were designated as being in a PCSA included Bear Valley Community Hospital, Kaiser Foundation Hospital - Fontana, Mountains Community Hospital, Colorado River Medical Center, Hi-Desert Medical Center, Desert Valley Hospital, and Barstow Community Hospital (Figure 1). San Bernardino County hospitals that were designated as being outside of a PCSA included Chino Valley Medical Center, Montclair Hospital Medical Center, San Antonio Regional Hospital, Community Hospital of San Bernardino, St. Bernardine Medical Center, and St. Mary Medical Center - Apple Valley (Figure 1). Among these hospitals, the highest rate of ED visits related to cardiac complications is seen in Desert Valley Hospital, where the percentage of cardiac-related ED visits was 0.49 (Figure 1).

**Figure 2:**
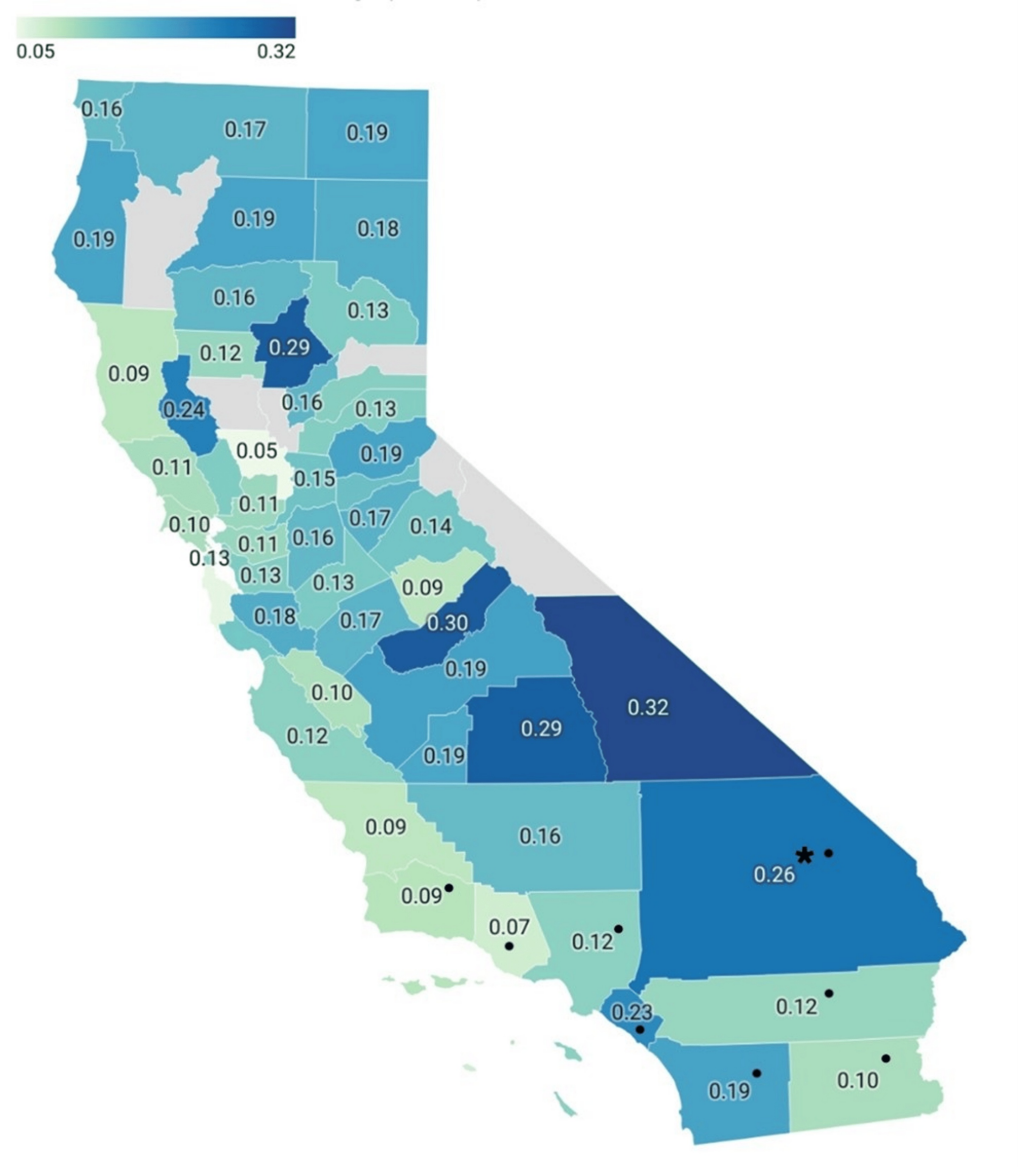
Percentage of ED visits related to cardiac complications in California per county (2021) Map of California depicting the average percentage of ED visits related to cardiac complications in 2021 per county. Gray-shaded areas indicate a county that was not included in the dataset. The star symbol indicates San Bernardino County. ● indicates counties of Southern California. Map: Lydia Wilson, Sasha Singh. Source: California Health and Human Services Open Data Portal. Created with Datawrapper.

Within Southern California, San Bernardino County had the highest rate of ED visits related to cardiac complications in 2021, with an average of 0.26% (Figure 2). However, it had the fifth-highest rate when compared to all of California (Figure 2).

Analyzing data by designation of PCSA

Results indicated that the average percentage of ED visits related to cardiac complications in San Bernardino’s PCSA hospitals (M = 0.285, SD = 0.124) are not statistically significant in their difference from San Bernardino hospitals that are located outside of PCSAs (M = 0.227, SD = 0.0960), t(11) = 0.928, p = 0.373 (Table 1). However, when analyzing data for all of Southern California, hospitals in PCSAs (M = 0.192, SD = 0.133) had significantly greater percentages of cardiac-related ED visits than hospitals not in a PCSA (M = 0.143, SD = 0.0969), t(119) = 2.197, p = 0.030 (Table 1). Similarly, when using data for all of California, PCSA hospitals (M = 0.192, SD = 0.120) also had a significantly higher percentage of ED visits related to cardiac complications than hospitals not in a PCSA (M = 0.135, SD = 0.0864), t(249) = 4.282, p = <0.001 (Table 1).

**Table 1:** Independent samples t-test comparing differences in the percentage of ED visits that are related to cardiac complications by designation of primary care shortage area

Region	Hospital in primary care shortage area			Hospital not in primary care shortage area			df	t	Two-sided p
	N	M	SD	N	M	SD			
San Bernardino	7	0.285	0.124	6	0.227	0.0960	11	-0.928	0.373
Southern California	30	0.192	0.133	91	0.143	0.0969	119	-2.197	0.030
California (statewide)	79	0.192	0.120	172	0.135	0.0864	249	-4.282	<0.001

Analyzing data by geographical region

Statistical analysis showed that hospitals in San Bernardino (M = 0.259, SD = 0.111) had significantly greater rates of ED visits that were related to cardiac complications when compared to hospitals in the rest of Southern California (M = 0.143, SD = 0.102), t(119) = 3.845, p = <0.001 (Table 2). Furthermore, hospitals in San Bernardino (M = 0.259, SD = 0.111) also had significantly greater rates of cardiac-related ED visits when compared to hospitals in the rest of California (M = 0.147, SD = 0.0981), t(249) = 3.963, p = <0.001 (Table 2).

**Table 2:** Independent samples t-test comparing the percentage of ED visits that are related to cardiac complications by geographical region

Region	N	M	SD	df	t	Two-sided p
San Bernardino	13	0.259	0.111	119	-3.845	<0.001
Southern California	108	0.143	0.102			
San Bernardino	13	0.259	0.111	249	-3.963	<0.001
California (statewide)	238	0.147	0.0981			

## Discussion

When considering Southern California and all of California, there was a significantly higher burden of cardiac cases on EDs in PCSAs compared to those in areas with adequate primary care accessibility. However, this difference was not observed when comparing hospitals only within San Bernardino County.

The significantly higher rate of cardiac-related ED visits in PCSA hospitals in Southern California and California likely reflects a lack of primary care follow-up for chronic heart conditions. Close follow-up with primary care providers is vital for managing cardiac conditions such as heart failure, coronary artery disease, and arrhythmias. For example, heart failure is a condition that can often be controlled with regular visits to a primary care provider or cardiologist through proper medication management. However, when not treated adequately, patients often experience acute exacerbations, which require an ED visit, usually followed by hospital admission [16]. While we cannot distinguish between different cardiac diagnoses in the data used for this study, we did find that hospitals in PCSAs had significantly higher rates of cardiac diagnoses in the ED. It is highly likely that many of these patients could have avoided going to the ED and the likely subsequent hospitalization if they had had access to a primary care provider to manage their chronic conditions.

Furthermore, with the increasing wait times and caseloads in EDs throughout California, it is vital to do everything possible to limit the need for patients with chronic conditions to be treated in an ED setting [10]. Treating chronic cardiac conditions in primary care settings would likely not only improve patient outcomes but would also likely lead to an overall reduction in the burden of cardiac cases in the ED and a decrease in the overall caseload.

It is important to note, however, that prior studies have found varying effects on decreasing ED utilization with increasing primary care access [17]. It is unclear where the discrepancy between these studies lies, but most of the literature indicates that increased primary care access has a positive effect on decreasing hospital admissions [17]. While we are primarily focused on evaluating the ED burden in this study, an overall decrease in hospital admissions for cardiac-related conditions is still a favorable outcome that could come from increased primary care access throughout California.

There are many possible reasons for the lack of a statistically significant difference between PCSA and non-PCSA hospitals in San Bernardino County, one being that data was available for only 13 hospitals, creating a small sample size that may not encapsulate the trends of the county. Additionally, there could be confounding factors unique to San Bernardino County that we did not account for. For example, our data showed that San Bernardino County has the fifth-highest frequency of cardiac-related ED cases in California. This increased number of patients could account for some of the increased cases in the ED, even in areas with adequate primary care coverage. Furthermore, as discussed above, a significant proportion of the population in San Bernardino County has low socioeconomic status, possibly associated with social determinants of health that may be affecting this regardless of access to primary care providers.

Regarding the overall ED burden of cardiac cases, when compared to the rest of Southern California, San Bernardino had significantly higher rates of ED visits that were related to cardiac complications in 2021. This correlates to data from the Centers for Disease Control and Prevention that shows San Bernardino as having the highest rate of heart disease hospitalizations, 38.3 per 1,000, in Southern California for patients 65 years and older between the years 2019 and 2021 [18]. Furthermore, San Bernardino’s rate of cardiac-related ED visits was shown to be significantly higher than that of all of California in 2021.

San Bernardino County is unique when considering access to healthcare and determinants of healthcare as it is the largest county in the contiguous United States [19]. As a whole, San Bernardino County fails to hit the national target ratio for patients per primary care provider, which could be a contributing factor to these findings [6]. However, this cannot fully explain the disparities between San Bernardino County and the rest of the state, as we found no significant difference between hospitals in a PCSA versus those that were not within San Bernardino County.

Therefore, we must consider the unique factors within San Bernardino County that could be contributing to the higher burden of cardiac cases in the ED. One such factor may be that San Bernardino County consistently has poor air quality when compared to other counties in California [20]. In 2021, the most common air quality status within the county was “moderate,” which indicates an AQI score of 51-100 [19]. Poor air quality is known to exacerbate chronic diseases, including many cardiac conditions, which indicates that this could be a contributing factor to the increased burden on the ED.

Another possible contributing factor may be that San Bernardino County has a higher proportion of residents living in poverty and a lower percentage of residents with health insurance when compared to all of California [6]. In 2019, 90.9% on San Bernardino County had health insurance compared with the statewide of 92.3% coverage [6]. Individuals living in poverty and/or without access to health insurance often are more susceptible to chronic health conditions, including cardiac issues, as they are unable to access important preventative measures.

There are many other possible factors that could contribute to the higher ED burden of cardiac cases in San Bernardino County compared to the rest of California, and that is an important target for future research. There is a lack of research on healthcare access and outcomes in San Bernardino County, and this study highlights the need for continued studies to better understand how to prevent the disparities that we are seeing.

The disparities shown in our findings highlight a need for increased preventative care and management of chronic conditions in primary care settings. A major barrier to accessing primary care is a lack of providers in rural areas. One way to mitigate this disparity would be to expand access to telemedicine, as this allows patients to maintain longitudinal care without the burden of having to travel to their provider. This would also likely help reduce the disparities seen between San Bernardino County and the rest of the state, as San Bernardino County has a relative shortage of primary care providers, and much of the county is rural.

Another intervention that would specifically target San Bernardino County is the implementation of legislation around improving public health. One probable contributing factor to the disparity that we found is the poor air quality as discussed above. By implementing legislation that limits pollutant release, there would likely be much fewer complications from cardiac and respiratory diseases.

This study had many limitations that also necessitate further research on this topic. One limitation is the lack of data for all hospitals in San Bernardino County, as stated previously. The current count of 13 hospitals created a small sample size for our statistical analysis and did not allow us to have a full picture of ED visits related to cardiac complications in the county. With this, we also recognize that not all hospitals in the state were accounted for in the data. It is unknown if data from these hospitals would have altered our results. Additionally, we do not know how the dataset determined ED visit classifications and do not have a definition for what it meant to have a cardiac-related ED visit. This would be helpful information to be able to distinguish between chronic and acute conditions. Finally, there was an absence of demographic information in this dataset; we do not have a reference for the age group, socioeconomic status, etc., for the patients included.

Future studies should incorporate more hospital data to gather a broader understanding of the burden of heart disease in EDs across California. Additionally, further research should incorporate demographic information of patients to provide information on possible underlying relationships.

## Conclusions

Our research showed that there is a significant correlation between the frequency of cardiac-related ED cases and primary care shortages. We also found that San Bernardino County has the fifth-highest rate of cardiac burden on ED cases in California, and this rate is significantly higher than that of the rest of Southern California and that of the rest of California. Some modifications to help alleviate these trends may be to increase access to telemedicine in rural areas as well as promote legislation to help reduce air pollution in the region. This points to a need for further studies on the factors contributing to exacerbations of cardiac conditions in San Bernardino County. It is also imperative to further study the ways that primary care shortages affect ED caseload and how we can mitigate this disparity.
